# MicroRNA-129-3p Suppresses Tumor Progression and Chemoradioresistance in Head and Neck Squamous Cell Carcinoma

**DOI:** 10.3390/curroncol32010054

**Published:** 2025-01-20

**Authors:** Tae Mi Yoon, Sun-Ae Kim, Eun Kyung Jung, Young-Kook Kim, Kyung-Hwa Lee, Sang Chul Lim

**Affiliations:** 1 Departments of Otorhinolaryngology-Head and Neck Surgery, Chonnam National University Medical School, Hwasun Hospital, Hwasun 58128, Jeonnam, Republic of Korea; yoontm@chonnam.ac.kr (T.M.Y.);; 2 Departments of Biochemistry, Chonnam National University Medical School, Hwasun Hospital, Hwasun 58128, Jeonnam, Republic of Korea; 3 Departments of Pathology, Chonnam National University Medical School, Hwasun Hospital, Hwasun 58128, Jeonnam, Republic of Korea

**Keywords:** miRNA, radioresistance, apoptosis, molecular targeted therapy, head and neck squamous cell carcinoma

## Abstract

(1) Background: MicroRNA-129 (miR-129) participates in tumor progression and chemoresistance in various cancer types. In this study, the role of miR-129-3p, the main mature form of miR-129, in tumor progression and chemoradiotherapy resistance in head and neck cancer was evaluated. (2) Methods: RT-PCR, western blotting, cell proliferation assays, cell apoptosis assays, and cell invasion and migration assays were used. (3) Results: In this study, the miR-129-3p overexpression suppressed the proliferation, invasion, and migration of SNU1041, SCC15, and SCC25 human HNSCC cell lines. Additionally, it induced apoptosis and enhanced radiation-/cisplatin-induced apoptosis of SNU1041, SCC15, and SCC25 cells. Therefore, miR-129-3p could suppress tumor progression and enhance chemoradiosensitivity in human HNSCC. Furthermore, miR-129-3p was downregulated in fresh tumor tissues from patients with HNSCC compared with that in the adjacent normal mucosa. In a nude mouse xenograft model with SNU15 cells, the miR-129-3p overexpression significantly decreased tumor growth, as measured by tumor weight and volume. (4) Conclusions: Our study provides evidence that miR-129-3p suppresses tumor progression and chemoradioresistance in HNSCC. This finding may serve as a basis for developing miR-129-3p-based therapeutic strategies.

## 1. Introduction

Head and neck cancer is the sixth-most common malignant tumor among various cancer types, accounting for approximately 5.3% of cases [1]. The most prevalent type of head and neck cancer is squamous cell carcinoma of the head and neck [2]. The incidence of head and neck cancer has been increasing over the past decade [3]. It is treated with surgery and chemoradiotherapy; however, patients with advanced head and neck cancer usually receive adjuvant radiotherapy or chemoradiotherapy, even if they undergo surgery [4]. Despite recent advancements in surgical techniques, chemotherapy, and radiotherapy, the prognosis for advanced head and neck cancer remains poor [5]. Because most patients with advanced head and neck cancer undergo chemoradiotherapy either before or after surgery, predicting and improving the response to treatment are necessary for improving the prognosis for these patients.

MicroRNAs (miRNAs) function as a key post-transcriptional regulator by inhibiting mRNA translation or degradation [6]. As such, they participate in regulating cell proliferation, apoptosis, and chemoresistance by acting as either oncogenes or tumor suppressors in tumor pathology [7,8]. Additionally, they are crucial in tumor metastasis by regulating epithelial–mesenchymal transition (EMT), invasion, and tumor cell self-renewal [9]. Among miRNAs, miRNA-129 (miR-129) is involved in the progression of various cancer types, and its expression correlates with patient survival [10,11,12]. Two miRNAs, miR-129-3p and miR-129-5p, are derived from opposite arms of the same precursor, miR-129, with miR-129 and miR-129-3p being the principal mature forms originating from this precursor [13]. While the link between miR-129 and cancer progression has been explored in several prominent cancers, such as breast, colorectal, and esophageal cancers [14,15,16], this study represents the first investigation into its role in head and neck cancer.

In this research, we examined the impact of miR-129-3p on tumor progression and chemoradioresistance in head and neck squamous cell carcinoma (HNSCC) cell and a xenograft model of nude mice. Our findings revealed that miR-129-3p is significantly downregulated in fresh tumor specimens from HNSCC patients compared to corresponding normal mucosa. This establishes a foundation for the development of therapeutic strategies centered on miR-129-3p.

## 2. Materials and Methods

### 2.1. Cell Culture and Transfection

The HNSCC cell lines SCC15 and SCC25 were obtained from the American Type Culture Collection (Manassas, VA, USA), while the SNU1041 cell line was sourced from the Korean Cell Line Bank (Seoul, Republic of Korea). SCC25 and SCC15 cells were cultured in DMEM/F12 medium (GIBCO^®^, Invitrogen, Carlsbad, CA, USA), whereas SNU1041 cells were maintained in RPMI medium (GIBCO^®^, Invitrogen) supplemented with 10% fetal bovine serum (FBS, GIBCO^®^, Invitrogen) and 1% penicillin/streptomycin in a 37 °C humidified incubator with 5% CO_2_.

To overexpress endogenous miR-129-3p in the HNSCC cells, a miR-129-3p mimic (Assay ID: MC10076, Cat. No. 4464066, Applied Biosystems; Thermo Fisher Scientific, Inc., Waltham, MA, USA) was utilized. The cells were seeded at a density of 2.0 × 10^5^ cells per well in 6-well plates and then transfected with 100 pmol of either the miR-129-3p mimic or a negative control miRNA mimic using Lipofectamine RNAiMAX (Invitrogen; Thermo Fisher Scientific, Inc.) for 48 h at 37 °C. Subsequent experiments were performed 48 h post-transfection.

### 2.2. RNA Isolation and Reverse Transcription (RT) Quantitative Polymerase Chain Reaction (qPCR)

RNA from the cells was conducted using TRIzol reagent (Invitrogen; Thermo Fisher Scientific, Inc.) following the manufacturer’s protocol. Reverse transcription (RT) was carried out with a TaqMan™ microRNA reverse transcription kit (Cat. No. 4366579, Applied Biosystems; Thermo Fisher Scientific, Inc.) under the following conditions: 30 min at 16 °C for one cycle, 30 min at 42 °C for one cycle, and 5 min at 85 °C for one cycle. The resultant cDNA was then amplified using TaqMan^®^ Universal Master Mix II (Cat. No. 4440040, Applied Biosystems; Thermo Fisher Scientific, Inc.), employing one cycle at 95 °C for 10 min followed by 40 cycles consisting of 15 s at 95 °C and 60 s at 60 °C. RNU44 served as the endogenous control. Primers for RNU44 and miR-129-3p were obtained from Thermo Fisher Scientific, Inc. (miR-129-3p, Assay ID: 001184; RNU44, Assay ID: 001094, Cat. No. 4427975).

RT-qPCR analysis was conducted using the QuantStudio 3 Real-Time PCR System (Cat. No. 34665; Applied Biosystems; Thermo Fisher Scientific, Inc). The expression of miR-129-3p was analyzed with the QuantStudio Design & Analysis Software v 1.3 (Thermo Fisher Scientific, Inc.). The Ct value of each miR-129-3p of normal tissues and tumor obtained after the experiment was compared to the Ct value of endogenous control. RT was performed prior to qPCR. Amplification plots were assessed to determine the quantification cycle (Cq), and each reaction was conducted independently a minimum of three times.

### 2.3. Protein Isolation and Western Blot Analysis

Cells were lysed using a radioimmunoprecipitation assay buffer (Biosesang Inc., Yongin-si, Republic of Korea), and protein concentrations were quantified using the bicinchoninic acid (BCA) assay. Following a 10–12% sodium dodecyl sulfate–polyacrylamide gel electrophoresis (SDS-PAGE), protein lysates (20–30 µg per lane) were separated and then transferred electrophoretically onto polyvinylidene fluoride membranes. The membranes were incubated at room temperature for one hour in Tris-buffered saline (TBS) with 0.1% Tween-20, supplemented with 5% bovine serum albumin (BSA) (Bioshop Canada Inc.). Afterward, the membranes were washed four times with TBS containing 0.1% Tween-20, with each wash lasting 15 min.

Specific proteins were detected using primary antibodies against β-actin (Cat. No. 3700; Cell Signaling Technology, Inc., Danvers, MA, USA), cleaved caspase-3 (Cat. No. 9664; Cell Signaling Technology, Inc.), cleaved caspase-7 (Cat. No. 9491; Cell Signaling Technology, Inc.), X-linked inhibitor of apoptosis protein (XIAP; Cat. No. sc-11426; Santa Cruz Biotechnology, TX, USA), and cleaved poly (ADP-ribose) polymerase (PARP; Cat. No. 5625; Abcam, UK). The primary antibodies were diluted to 1:1000 in TBS with 0.1% Tween-20 and incubated with the membranes at 4 °C for 24 h.

Secondary antibodies (anti-rabbit, Cat. No. 7074; Cell Signaling Technology, Inc. or anti-mouse, Cat. No. 7076; Cell Signaling Technology, Inc.) conjugated to horseradish peroxidase (HRP) were diluted 1:2000 and incubated with the membranes at room temperature for 2 h. Immunoreactive proteins were visualized using an enhanced chemiluminescence detection system for HRP (EMD Millipore, Burlington, MA, USA) and analyzed with an LAS 4000 luminescent image analyzer (FUJIFILM Wako Pure Chemical Corporation, Osaka, Japan). Western blot analysis was performed independently in three separate experiments.

### 2.4. Cell Proliferation Assay

The cells were seeded in 24-well plates at a density of 1 × 10^4^ cells per well and transfected the following day. After 48 h of culture, cell viability was assessed using the EZ-CyTox (tetrazolium salts, WST-1) enhanced cell viability assay kit (Cat. No. EZ-3000; Daeil Lab Inc., Yongin-si, Republic of Korea) at 37 °C for 1 to 2 h. Absorbance was measured at 460 nm using a microplate reader. Cell viability assays were performed independently in triplicate.

### 2.5. Cell Invasion Assay

Cell invasion was evaluated by counting the number of cells that migrated through an 8.0 µm pore Transwell invasion device (Cat. No. 3422; Costar, Inc., Washington, DC, USA). The upper chamber was coated with a 1% gelatin solution for 12 h at 37 °C and allowed to dry at room temperature for an additional 12 h, 1 day before the experiment. After a 48 h transfection period, the upper chamber was seeded with 2 × 10^5^ cells in 120 µL of 0.2% bovine serum albumin (BioShop Canada, Inc., Burlington, ON, Canada) in FBS-free DMEM. For the lower chamber, 400 µL of 0.2% bovine serum albumin (BioShop Canada, Inc.) in FBS-free DMEM containing fibronectin (Cat. No. 361635; EMD Millipore) was added as a chemoattractant.

Following 24 h of incubation, the cells that had migrated to the bottom surface of the Transwell were stained using a Diff-Quik solution (Sysmex Corporation, Kobe, Japan). The cells were then counted in five random microscopic fields at 100× magnification using an optical microscope. Results are expressed as the mean number of cells per field ± standard error from three independent experiments.

### 2.6. Cell Migration Assay (Wound Healing Assay)

The transfected cells were plated at a density of 1.5 × 10^5^ cells per well in each well of Culture-Inserts (Ibidi GmbH, Gräfelfing, Germany). After 24 h of incubation, the inserts were removed, and imaging was conducted at 0, 4, 8, and 12 h using an inverted microscope to observe the progress of cell migration. The distances between the gaps were normalized to 1 cm after capturing images from three random sites.

### 2.7. Apoptosis Assay

Apoptosis was evaluated using an Annexin V–fluorescein isothiocyanate (FITC) assay. Following a 48 h transfection period, cells were collected by trypsinization, washed twice with phosphate-buffered saline, and then resuspended in a binding buffer (BD Biosciences, Franklin Lakes, NJ, USA). Annexin V-FITC and 7-amino-actinomycin D (BD Biosciences) were added to the cell suspension, which was then incubated in the dark for 15 min before being resuspended in 400 µL of binding buffer.

The samples were analyzed using a FACSCalibur flow cytometer (BD Biosciences) and BD Cell Quest version 3.3 software (Becton Dickinson, Franklin Lakes, NJ, USA). Data analysis was performed using WinMDI version 2.9 (The Scripps Research Institute, La Jolla, CA, USA). Apoptosis assay experiments were conducted independently in triplicate.

### 2.8. Cell Irradiation or Cisplatin Treatment

After 48 h of transfection, the cells were cultured at 37 °C and exposed to γ-irradiation at room temperature using a Gammacell 3000 Elan (Therathronics, Ottawa, ON, Canada) at various doses of 10 and 20 Gy (137Cs, 2.875 Gy/min). A stock solution of cisplatin (10 mg/20 mL; Dong-A, Co., Ltd., Seoul, Republic of Korea) was prepared in each medium and diluted to different concentrations (5 and 10 μg/mL) for experimental use at 37 °C for 24 h.

### 2.9. Patients and Tumor Specimens

Fresh tumor and adjacent normal tissues were acquired from 16 patients (8 patients with oral cancer; 2 of stage I, 3 of stage II, 3 of stage III/8 patients with hypopharyngeal cancer; 2 of stage II, 4 of stage III, 2 of stage IV) who underwent surgery for HNSCC at Chonnam National University Hwasun Hospital (Jeonnam, Republic of Korea) between September 2005 and December 2015 to analyze miRNA-123-3p expression. The tumor and adjacent normal tissues were reviewed by two independent pathologists and were diagnosed to HNSCC and normal tissues through histopathologic examination. The collected tissues were sourced prior to any radiotherapy and/or chemotherapy. All patients provided written informed consent for the use of their resected tissue samples.

### 2.10. Nude Mouse Xenograft Model

Mice were randomly assigned to a control group (*n* = 10) and a miR-129-3p mimic group (*n* = 10) to evaluate the anti-tumor growth effects of miR-129-3p. SCC15 cells were transfected with a miR-129-3p mimic, and 48 h post-transfection, either the miR-129-3p mimic-transfected or control SCC15 cells (1 × 10^6^ cells in 100 µL of serum-free media) were injected subcutaneously into the dorsal regions of 6-week-old female nude mice (Orient Bio Inc., Seognam-si, Gyeonggi-do, Republic of Korea). Tumor size was measured every 5 days until the mice were sacrificed on day 25. On day 25, tumors were excised, and tumor weight was concurrently recorded. All animal procedures were approved by the Chonnam National University Medical School Research Institutional Animal Care and Use Committee (IACUC; CNU IACUC-H-2020-2).

### 2.11. Statistical Analysis

The experimental differences were assessed using an unpaired Student’s *t*-test. Data are expressed as mean ± standard error. All experimental analyses were carried out independently in triplicate. Statistical analyses were performed using SPSS version 21.0 (IBM, Corp., Armonk, NY, USA), with *p* < 0.05 considered statistically significant.

## 3. Results

### 3.1. miR-129-3p Overexpression Suppresses Tumor Cell Proliferation, Invasion, and Migration in Human HNSCC Cells

**Figure 1:**
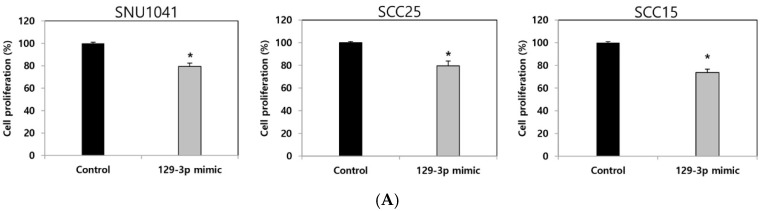

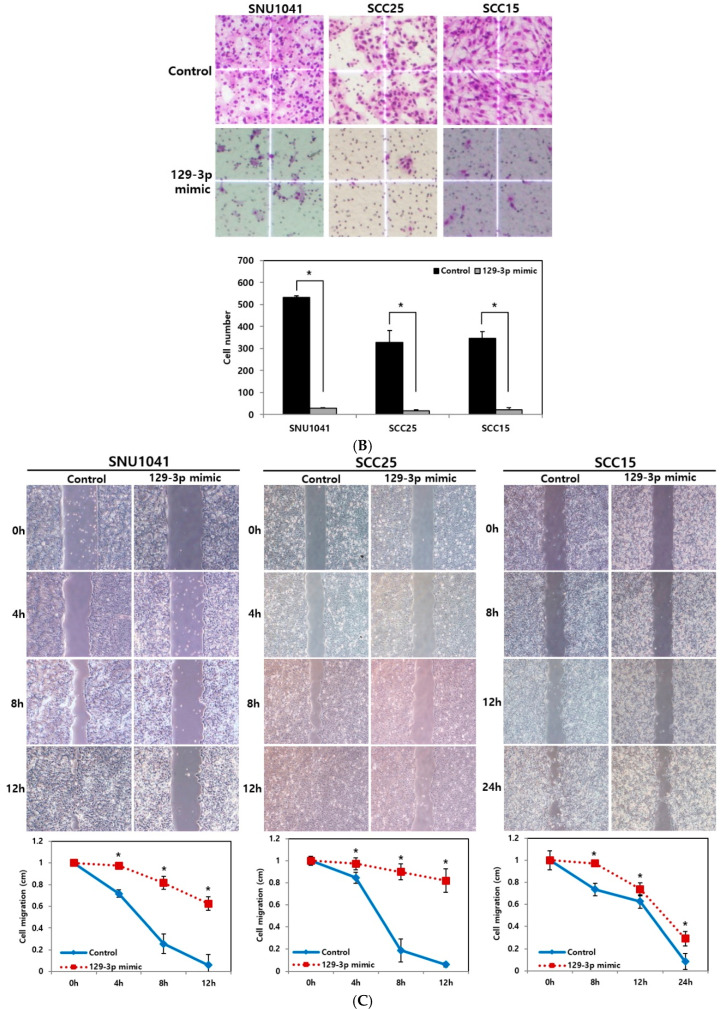
Effect of the miRNA-129-3p overexpression on cell proliferation, invasion, and migration in human HNSCC cells. (**A**) miR-129-3p mimic-transfected HNSCC cells significantly suppressed the proliferation in the SNU1041, SCC25, and SCC15 cells compared with that in the control cell. (**B**) Significantly fewer miR-129-3p mimic-transfected SNU1041, SCC25, and SCC15 cells demonstrated the invasion capacity compared with the control cells in a cell invasion assay. Magnification: ×100. The stained invading cells were counted and presented as the mean ± standard error in three independent experiments. (**C**) The artificial wound gap in the control cells in the cell migration assay became significantly narrower than that in the miR-129-3p mimic-transfected SNU1041, SCC25, and SCC15 cells. Cell migration is displayed as relative healing distances measured in three random sites. Values indicate mean ± SE for three independent experiments. 129-3p mimic, miR-129-3p mimic-transfected cells, * *p* < 0.05 vs. control.

In the present study, the role of miR-129-3p in tumor progression was investigated using miR-129-3p mimic to overexpress the endogenous expression of miR-129-3p in SNU1041, SCC25, and SCC15 human HNSCC cells. In the cell proliferation assay, the miR-129-3p mimic-transfected HNSCC cells significantly suppressed the proliferation of the SNU1041, SCC25, and SCC15 cells compared with the control cells (*p* < 0.05; Figure 1A). In the cell invasion assay, the invasion of miR-129-3p mimic-transfected HNSCC cells was significantly inhibited in all three cell lines (*p* < 0.05; Figure 1B).

In the cell migration assay, the artificial wound gap became significantly wider in the plates of the miR-129-3p mimic-transfected cells compared with that of the control SNU1041, SCC25, and SCC15 cells at 4, 8, and 12 h (*p* < 0.05; Figure 1C). The wound gaps in the plates of miR-129-3p mimic-transfected SNU1041 and SCC25 cells remained unfilled and noticeably wide even after 12 h. The miR-129-3p overexpression inhibited the cell migratory ability in SNU1041, SCC25, and SCC15 cells.

### 3.2. miR-129-3p Overexpression Induces Tumor Cell Apoptosis and Enhances Chemoradiosensitivity in Human HNSCC Cells

**Figure 2:**
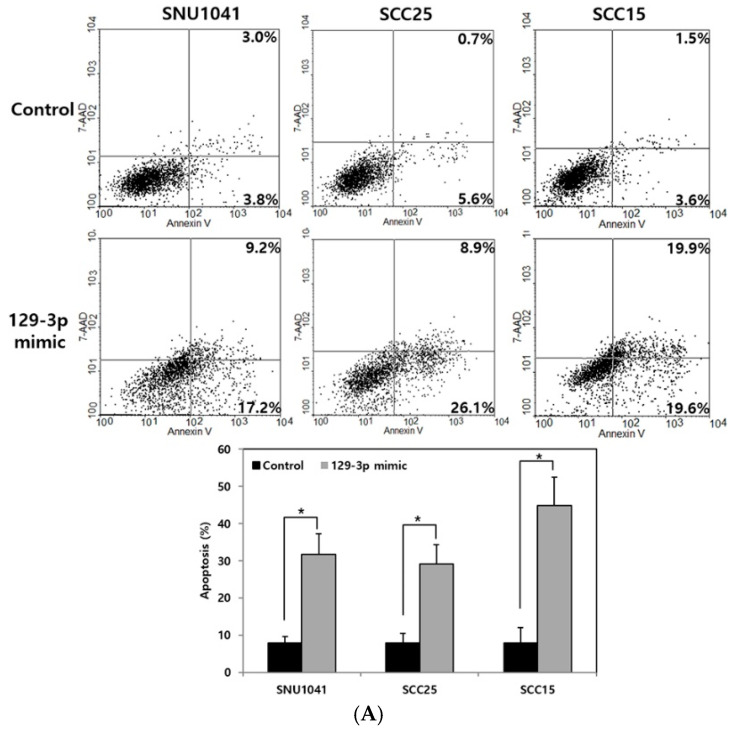

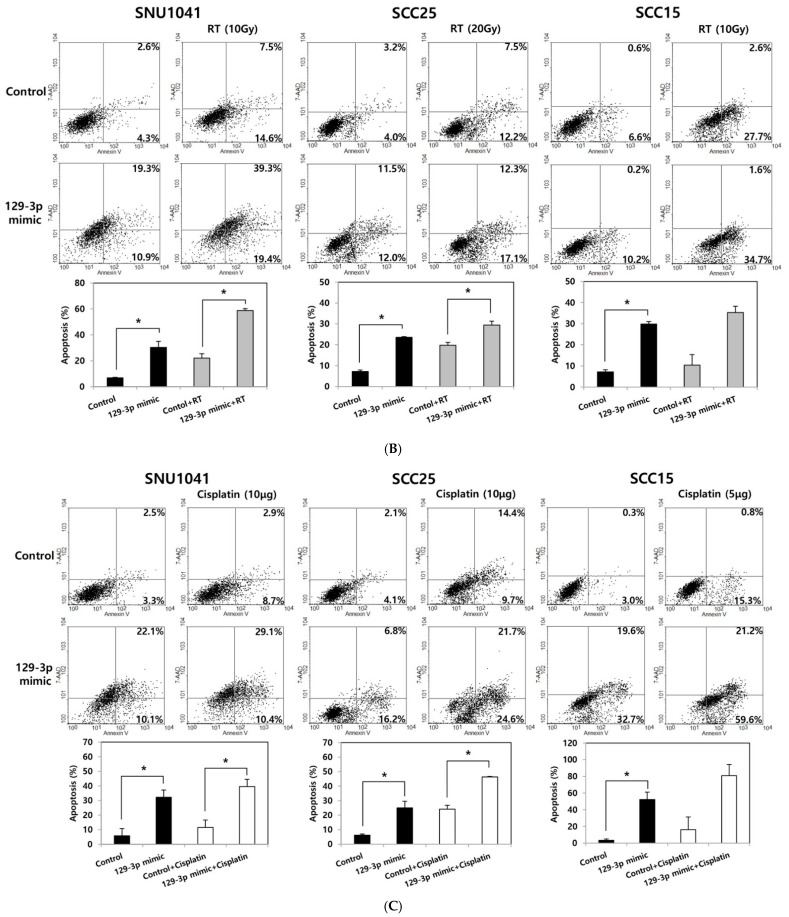

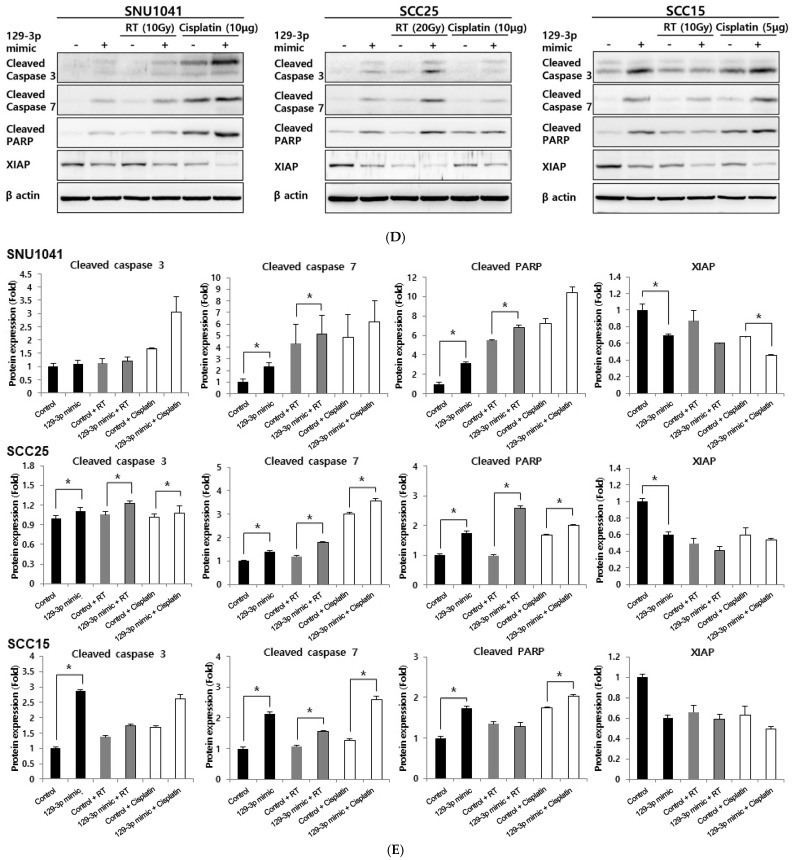
Effect of the miRNA-129-3p overexpression on the apoptosis and chemoradioresistance of human HNSCC cells. (**A**) In a cell apoptosis assay, flow cytometry demonstrated that the apoptosis of miR-129-3p mimic-transfected SNU1041, SCC25, and SCC15 cells was greater than that of the control cells. (**B**) The combination treatment of miR-129-3p mimic-transfected SNU1041 and SCC25 with 10 or 20 Gy radiation resulted in significantly greater apoptosis than the control treatment (radiation alone). (**C**) The combination treatment of miR-129-3p mimic-transfected SNU1041 and SCC25 with 10 μg/mL cisplatin resulted in significantly greater apoptosis than the control treatment (cisplatin alone). (**D**) The levels of cleaved caspase-3, cleaved caspase-7, and cleaved PARP were higher in the miR-129-3p mimic-transfected SNU1041, SCC25, and SCC15 cells than in the control cells. Furthermore, the expression levels of cleaved caspase-3, cleaved caspase-7, and cleaved PARP in the miR-129-3p mimic-transfected cells with the combination treatment (radiation or cisplatin) were greater than those in the control cells with radiation or cisplatin alone. (**E**) The intensity ratio of each band in the Western blot (**D**) was presented by densitometry analysis. The uncropped blots are shown in the Supplementary Materials. * *p* < 0.05 vs. control. 129-3p mimic, miR-129-3p mimic-transfected cells; 7-AAD, 7-amino-actinomycin D; RT, radiation therapy; PARP, poly(ADP-ribose)polymerase; XIAP, X-linked inhibitor of apoptosis protein.

An Annexin-V apoptosis assay was used to evaluate the effect of miR-129-3p on apoptosis. Flow cytometry revealed that the miR-129-3p overexpression significantly increased the proportion of apoptotic cells in the SNU1041, SCC25, and SCC15 human HNSCC cells (*p* < 0.05; Figure 2A).

Next, it was further examined whether the overexpression of the miR-129-3p enhances chemoradiosensitivity by inducing apoptosis in HNSCC cells. Radiation (10 and 20 Gy) or cisplatin (5 and 10 μg/mL) was applied to the cells after transfection with miR-129-3p mimic for 48 h. The apoptosis of SNU1041 and SCC25 was significantly higher in the combination of miR-129-3p mimic and radiation than in radiation alone (*p* < 0.05; Figure 2B). Consistently, the apoptosis of SNU1041 and SCC25 cells was significantly more pronounced in the combination group of miR-129-3p mimic and cisplatin than in cisplatin alone (*p* < 0.05; Figure 2C). Consistent with the flow cytometry result, after radiation or cisplatin treatment, the expression level of cleaved caspase-3, cleaved caspase-7, and cleaved PARP were significantly higher in the miR-129-3p-overexpressed cells compared to the control cells, while XIAP levels were lower (*p* < 0.05, Figure 2D,E). The uncropped whole Western blot showing all the bands with molecular weight markers, as shown in Figure 2D, is provided in the Supplemental Materials. These results suggested that the miR-129-3p overexpression improved chemoradiosensitivity by enhancing the apoptosis of human HNSCC.

### 3.3. miR-129-3p Overexpression Elicits an Anti-Tumor Effect on the Nude Mouse Xenograft Model

**Figure 3:**
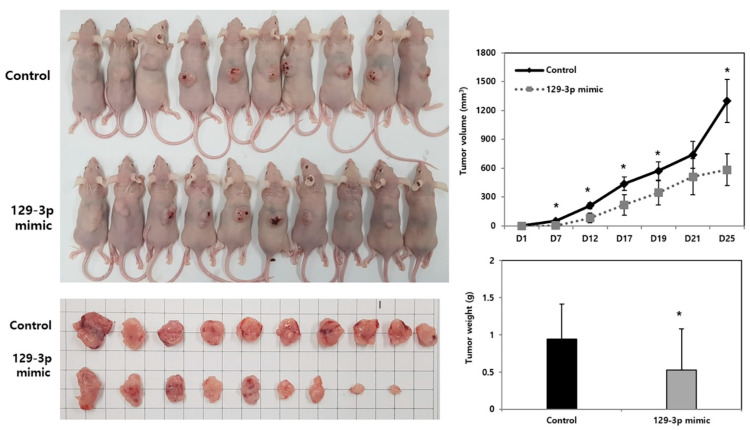
Anti-tumor effect of miRNA-129-3p overexpression on the nude mouse xenograft model. The tumors in the miR-129-3p group showed significantly slower growth in terms of volume and weight. * *p* < 0.05 vs. control. 129-3p mimic, miR-129-3p mimic-transfected cells implanted group.

miR-129-3p mimic-transfected or control SCC15 cells were implanted to the back of the nude mice to verify the anti-tumor growth inhibitory effect of miR-129-3p in vivo. The tumors in the miR-129-3p group consistently showed significantly slow growth in terms of tumor volume compared with control group from day 7 to day 25 (Figure 3). In addition, the weight of the tumor on day 25 in the miR-129-3p group was significantly lower than that in the control group (Figure 3). These results demonstrated the anti-tumor effect of miR-129-3p on the HNSCC nude mouse xenograft model.

### 3.4. miR-129-3p Expression in the Clinical Tumor Tissue and Normal Mucosa of Patients with HNSCC

**Figure 4:**
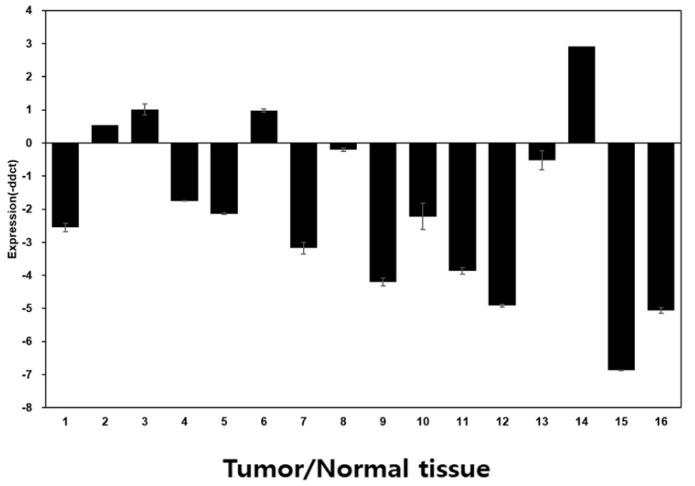
miR-129-3p expression in the tumor tissues of patients with HNSCC. Of the 16 patients, 12 (75%) showed a lower miR-129-3p expression in the tumor tissues than in the normal mucosa.

Fresh tumor and normal adjacent tissues from 16 patients were collected to evaluate the miR-129-3p expression. Of these patients, 12 (75%) showed a lower miR-129-3p expression in the tumor tissues than in the normal adjacent mucosa (Figure 4).

## 4. Discussion

Despite recent advancements in surgical techniques, radiation therapy, and chemotherapy, the prognosis for advanced head and neck cancer remains poor [5]. For this reason, tumor metastasis should be controlled to improve outcomes in head and neck cancer [5]. miRNAs play a key role in tumor metastasis by regulating processes such as EMT, tumor invasion, and tumor cell self-renewal [7,8,9]. Several miRNAs can function as oncogenes or tumor suppressors in HNSCC [17]. They are more stable than mRNAs in body fluids; as such, they are potential non-invasive biomarkers for patients with cancer [17]. Therefore, identifying miRNAs that can predict cancer progression and respond to chemoradiotherapy in HNSCC is crucial for improving treatment outcomes.

miR-129 is associated with various human cancers, including colorectal, bladder, endometrial, gastric, lung, and esophageal cancers [18,19,20,21,22,23]. In patients with colorectal cancer, the expression of miR-129-3p is significantly reduced in tumor tissues, and its overexpression inhibits cell proliferation, colony formation, migration, and tumor growth in colorectal cancer cell lines [24]. Similarly, this study observed that the miR-129-3p expression in HNSCC tumor tissues decreased, and the miR-129-3p overexpression in three different HNSCC cell lines inhibited cell proliferation, invasion, and migration. Additionally, miR-129-3p was confirmed to inhibit tumor growth in an in vivo nude mouse xenograft model of HNSCC. This finding suggested that miR-129-3p might function as a tumor suppressor in HNSCC. Therefore, it could be used as a biomarker for disease diagnosis and prognosis.

Several molecular mechanisms have been reported to be involved in the tumor suppressor function of miR-129-3p. miR-129-3p directly targeted sex-determining region Y-related high-mobility group-box 4 (*SOX4*), leading to its downregulation and inhibiting cell proliferation, migration, invasion, and EMT in colorectal and esophageal cancer cells [15,25]. In addition, it was associated with the activation of nuclear factor (NF)-kB signaling pathway [25]. *SOX4* is a transcription factor involved in embryonic development and cell differentiation, and its function as an oncogene in various cancers has been reported. In particular, SOX4 has been reported to be overexpressed in HNSCC and to be related to cancer progression and chemoradioresistance [26], so it might be worth investigating the relationship between miRNA-129-3p and *SOX4* in HNSCC through further research. *SOX4* has also been reported to regulate DNA damage repair and promote cisplatin and radiation resistance in lung adenocarcinoma and medulloblastoma [27,28]. This could be a potential mechanism by which miR-129-3p modulates cisplatin chemoradioresistance. Additionally, miR-129-3p negatively regulated Basic leucine zipper and W2 domains 1 (*BZW1*) functioned as cell cycle regulator, resulting in reduced cell proliferation in colorectal and ovarian cancer [24,29]. Furthermore, it has been reported that miR-129-3p targets Wide-type p53-induced phosphatase 1 (*Wip1*), E2F transcription factor 5 (*E2F5*), and Centromere coiled-coil protein 110 (*CCP110*) [14,30,31]. Further research is necessary to explore the target genes involved in the tumor suppressor function of miR-129-3p in HNSCC, as this could contribute to the establishment of novel therapeutic strategies targeting miR-129-3p in the future.

The most relevant achievement of this study is the validation that miR-129-3p can be used as a biomarker for predicting and overcoming resistance to cisplatin-based chemotherapy and/or RT. Cisplatin is a main chemotherapy agent widely used in HNSCC, and sensitivity to cisplatin is crucial for improving treatment outcomes. This agent forms DNA adducts that lead to apoptosis. Therefore, it is important to identify miRNAs that influence the apoptotic pathways in HNSCC and affect response to cisplatin. Moreover, since cisplatin-based concurrent chemoradiotherapy (CCRT) is the primary treatment method for advanced HNSCC, the implications of this study are remarkable. This study demonstrated that the miR-129-3p overexpression markedly inhibited apoptosis in HNSCC cells, thereby enhancing the therapeutic effects of cisplatin and RT by inducing apoptosis through apoptotic regulatory proteins such as caspase-3,7, PARP, and XIAP. This finding suggested that determining the miR-129-3p expression in clinical settings could help identify patients who are likely to respond well to CCRT; ultimately, treatment outcomes could be improved. Additionally, this study might be used as a basis for exploring combination therapy strategies alongside CCRT that would target miR-129-3p, which holds clinical importance.

However, this study has several limitations. First, although the study demonstrates that miR-129-3p has significant antitumor effects, the specific molecular pathways through which miR-129-3p exerts its effects were not fully elucidated. Understanding these mechanisms could provide deeper insights into its role and therapeutic potential. As mentioned above, *SOX4*, a target gene of miRNA-129-3p associated with chemoradioresistance in HNSCC, would be a valuable subject for future studies. Second, while the antitumor effect of miR-129-3p was observed in the mouse xenograft model, the impact on the effects of cisplatin and radiotherapy was not addressed. Future research is needed to explore the role of miR-129-3p in the context of chemoradiotherapy using an orthotopic model of head and neck cancer. The orthotopic model of HNSCC could more effectively replicate the characteristics of HNSCC, including its impact on diet, respiration, and cervical lymph node metastasis, which serve as critical prognostic factors in HNSCC. This model could hold potential for providing more accurate insights into the survival-extending effects of miR-129-3p. Third, the clinical sample size was small, limiting our ability to analyze treatment response and its association with clinical factors, including age, sea, smoking history, tumor location, and stage. It is necessary to explore the clinical significance of miR-129-3p in a larger cohort. These limitations will provide valuable suggestions for further research.

The next step for the clinical application of this study involves addressing the challenges related to the in vivo delivery method of miR-129-3p. For miR-129-3p to be utilized for therapeutic purposes of HNSCC, it is essential to establish an appropriate delivery method and determine the accurate dosage. miRNAs are prone to degradation in their natural state and face difficulties in crossing the cell membrane, leading to the development of various delivery methods [32]. Nanoparticle-based delivery systems, including liposomes, polymer nanoparticles, and gold nanoparticles, have been reported [32]. Additionally, viral vectors such as lentivirus, retrovirus, and adenovirus have been used to deliver miRNAs into cells [33]. However, these viral vectors may induce immune responses, necessitating further research to overcome this issue. It is also crucial to identify the optimal dosage of miR-129-3p for treating HNSCC. High doses of miRNA may induce toxicity, which has led to studies exploring sustained release systems, such as nanoparticles [32]. Furthermore, the development of targeting peptides that can selectively deliver miR-129-3p to HNSCC cells could enhance therapeutic efficacy while reducing the required dosage.

In summary, our study indicated that miR-129-3p inhibits tumor proliferation, invasion, and migration in HNSCC while inducing apoptosis; consequently, it suppresses tumor growth and enhances sensitivity to cisplatin and RT. Although further research may be needed, miR-129-3p expression may serve as an important biomarker for the diagnosis and treatment of HNSCC. Restoring this expression may be considered a novel therapeutic strategy to increase sensitivity to cisplatin-based CCRT.

## Data Availability

The datasets used and/or analyzed during the current study are available from the corresponding author upon reasonable request.

## Supplementary Materials

The following supporting information can be downloaded at: https://www.mdpi.com/article/10.3390/curroncol32010054/s1, the uncropped whole Western blot showing all the bands with molecular weight markers, as shown in Figure 2D.
