# Supplementary figures and images for: MicroRNA-129-3p Suppresses Tumor Progression and Chemoradioresistance in Head and Neck Squamous Cell Carcinoma
Source: Curr Oncol. 2025 Jan 20;32(1):54. doi: 10.3390/curroncol32010054 (PMC11763343; doi:10.3390/curroncol32010054)

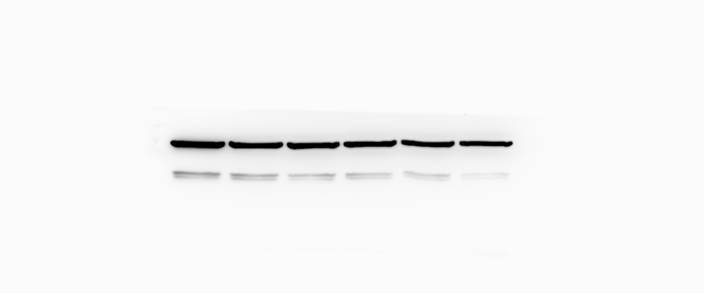

Supplement: Supplementary file 1 [file curroncol-32-00054-s001.zip › Supplemental materials_SCC15-BETA ACTIN.jpg]

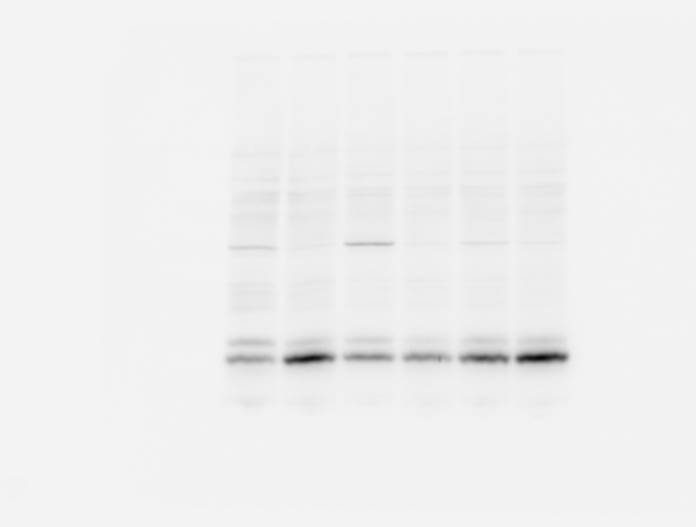

Supplement: Supplementary file 1 [file curroncol-32-00054-s001.zip › Supplemental materials_SCC15-Cleaved caspase3.jpg]

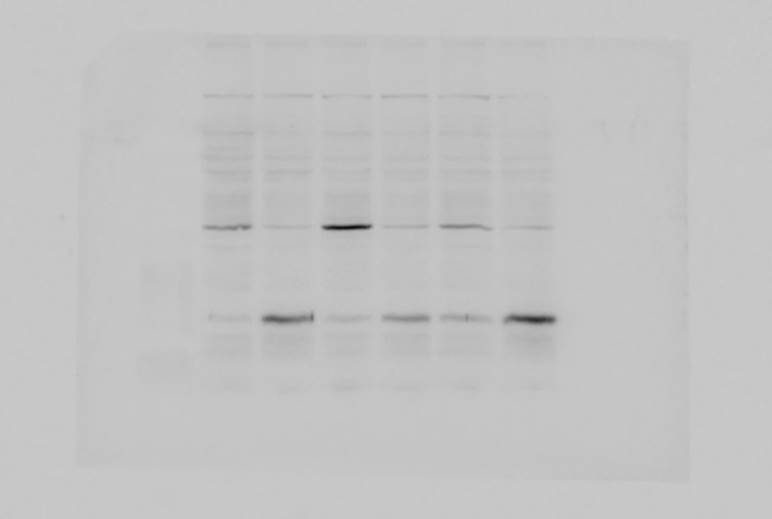

Supplement: Supplementary file 1 [file curroncol-32-00054-s001.zip › Supplemental materials_SCC15-Cleaved caspase7.jpg]

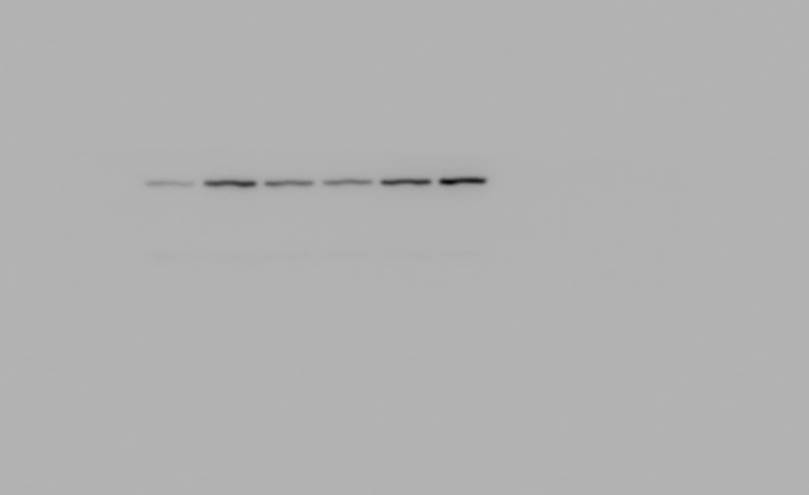

Supplement: Supplementary file 1 [file curroncol-32-00054-s001.zip › Supplemental materials_SCC15-Cleaved PARP.jpg]

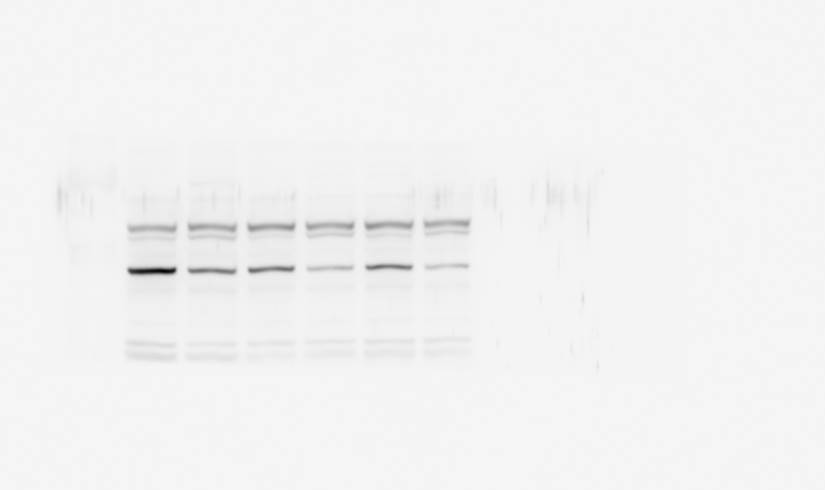

Supplement: Supplementary file 1 [file curroncol-32-00054-s001.zip › Supplemental materials_SCC15-XIAP.jpg]

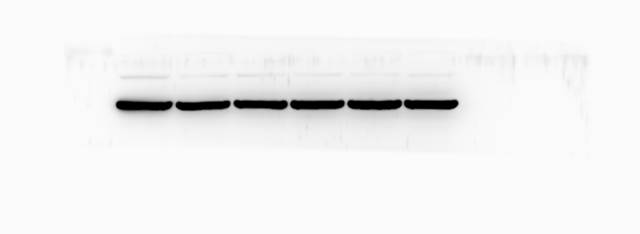

Supplement: Supplementary file 1 [file curroncol-32-00054-s001.zip › Supplemental materials_SCC25-BETA ACTIN.jpg]

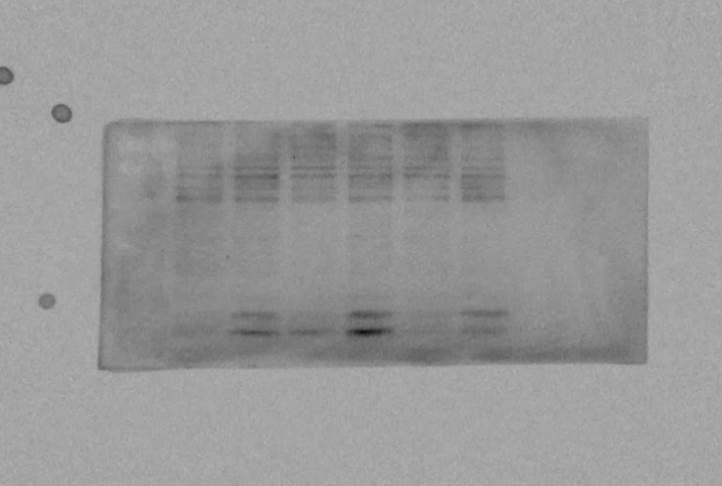

Supplement: Supplementary file 1 [file curroncol-32-00054-s001.zip › Supplemental materials_SCC25-Cleaved caspase3.jpg]

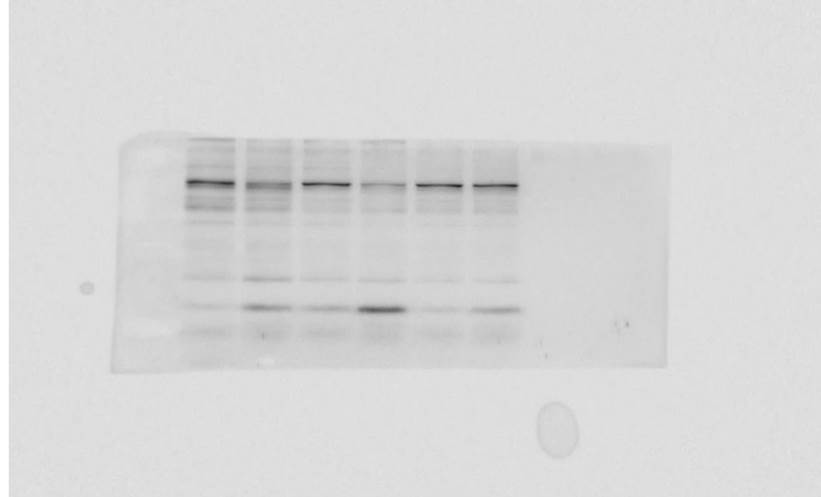

Supplement: Supplementary file 1 [file curroncol-32-00054-s001.zip › Supplemental materials_SCC25-Cleaved caspase7.jpg]

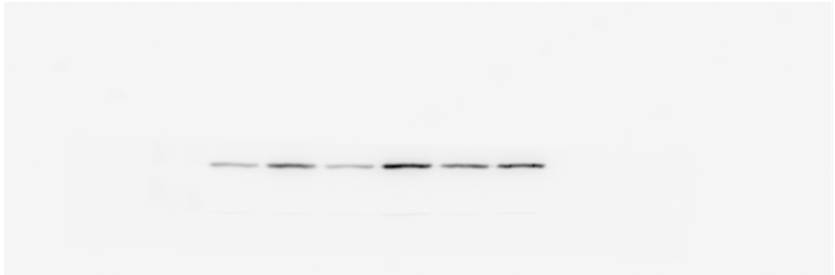

Supplement: Supplementary file 1 [file curroncol-32-00054-s001.zip › Supplemental materials_SCC25-Cleaved PARP.jpg]

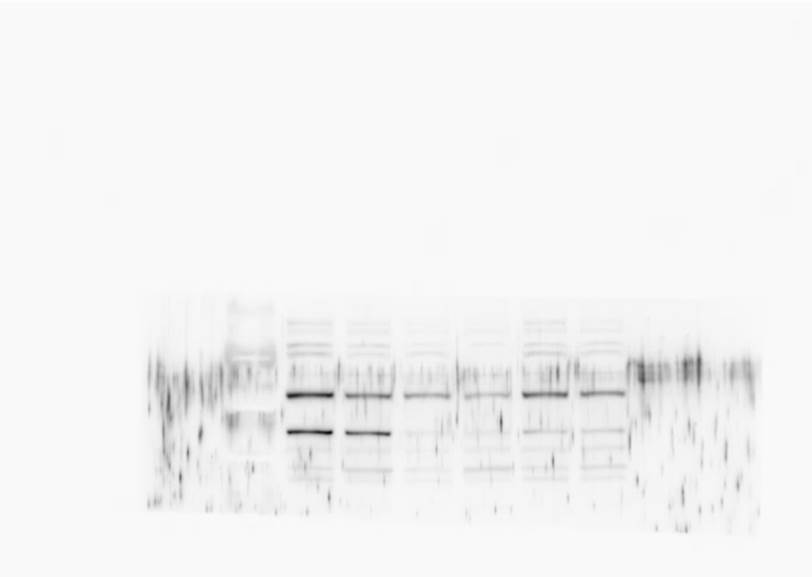

Supplement: Supplementary file 1 [file curroncol-32-00054-s001.zip › Supplemental materials_SCC25-XIAP.jpg]

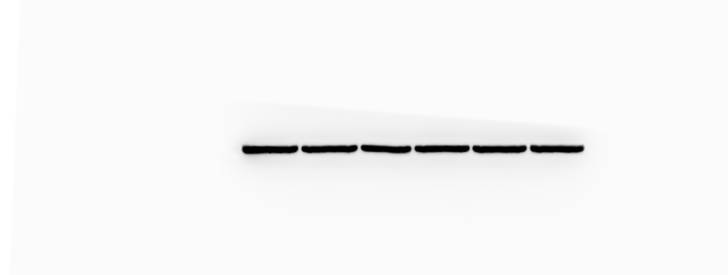

Supplement: Supplementary file 1 [file curroncol-32-00054-s001.zip › Supplemental materials_SNU1041-BETA ACTIN.jpg]

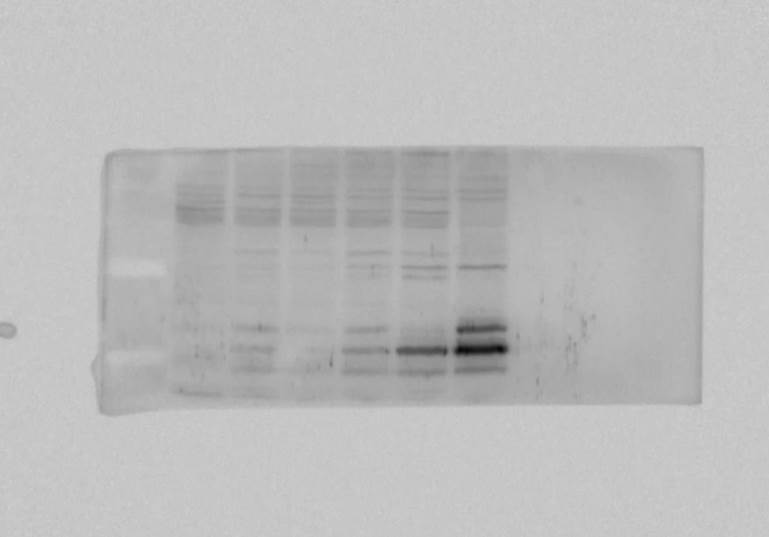

Supplement: Supplementary file 1 [file curroncol-32-00054-s001.zip › Supplemental materials_SNU1041-Cleaved caspase3.jpg]

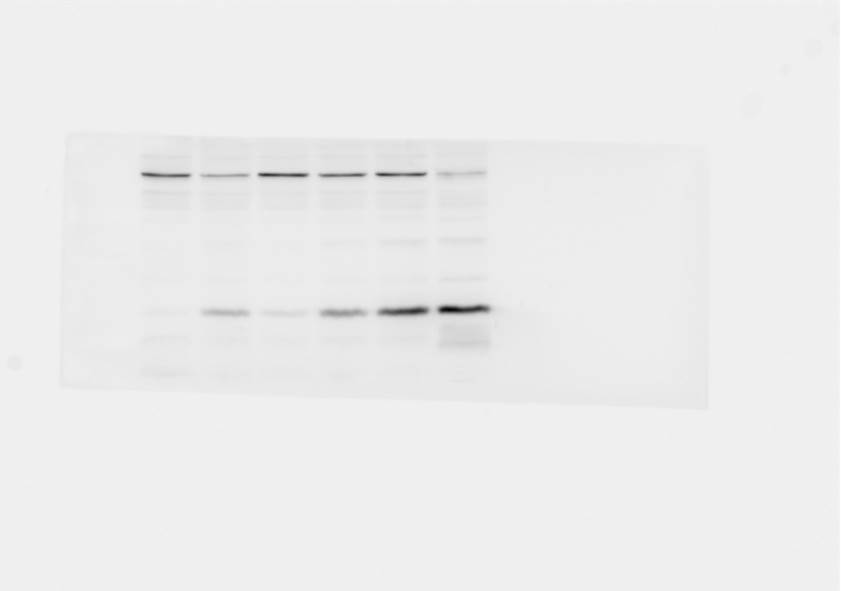

Supplement: Supplementary file 1 [file curroncol-32-00054-s001.zip › Supplemental materials_SNU1041-Cleaved caspase7.jpg]

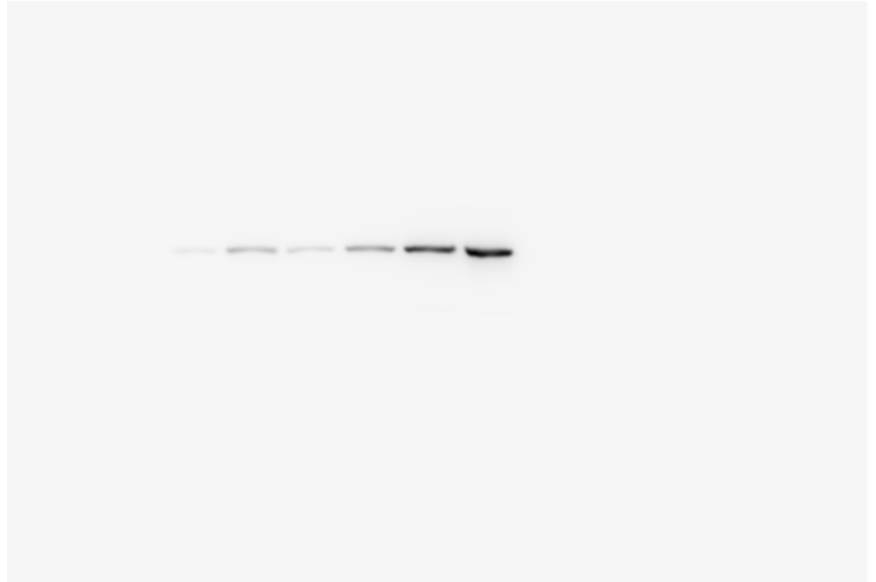

Supplement: Supplementary file 1 [file curroncol-32-00054-s001.zip › Supplemental materials_SNU1041-Cleaved PARP.jpg]

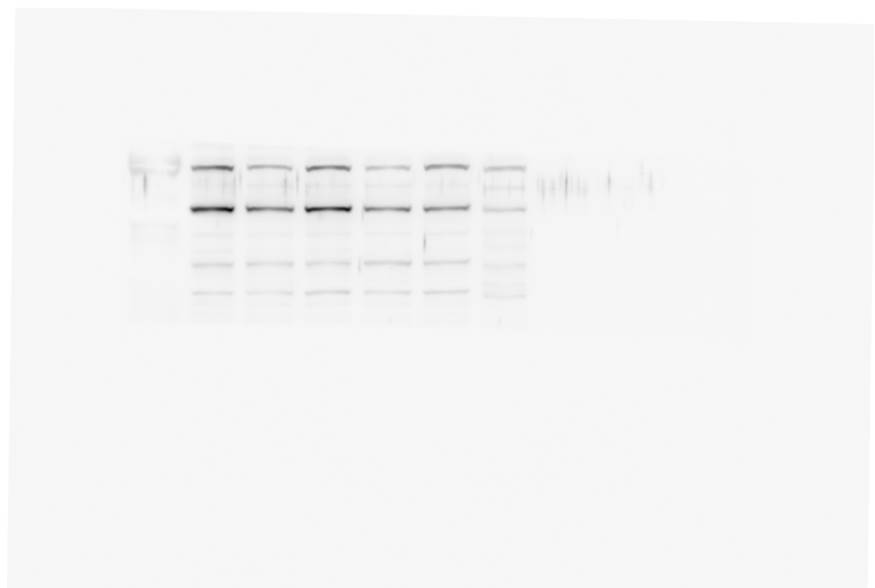

Supplement: Supplementary file 1 [file curroncol-32-00054-s001.zip › Supplemental materials_SNU1041-XIAP.jpg]
